# Ecological response hides behind the species abundance distribution: Community response to low‐intensity disturbance in managed grasslands

**DOI:** 10.1002/ece3.3395

**Published:** 2017-09-12

**Authors:** Atte Komonen, Merja Elo

**Affiliations:** ^1^ Department of Biological and Environmental Sciences University of Jyväskylä Jyväskylä Finland

**Keywords:** biodiversity, Carabidae, community assembly, Formicidae, Heteroptera, meadow, pasture, road verge, species abundance distributions

## Abstract

Land‐use and management are disturbance factors that have diverse effects on community composition and structure. In traditional rural grasslands, such as meadows and pastures, low‐intensity management is maintained to enhance biodiversity. Maintenance of road verges, in turn, creates habitat, which may complement traditional rural grasslands. To evaluate the effect of low‐intensity disturbance on insect communities, we characterized species abundance distributions (SAD) for Carabidae, Formicidae, and Heteroptera in three grassland types, which differed in management: meadows, pastures, and road verges. The shape of SAD was estimated with three parameters: abundance decay rate, dominance, and rarity. We compared the SAD shape among the grassland types and tested the effect of environmental heterogeneity (plant species richness) and disturbance intensity (trampling in pastures) on SADs. The shape of SADs did not differ among the grassland types but among the taxonomic groups instead. Abundance decay rate and dominance were larger for Formicidae, and rarity smaller, than for Carabidae and Heteroptera. For Carabidae and window‐trapped Heteroptera, rarity increased with increasing plant species richness. For Formicidae, dominance increased with trampling intensity in pastures. Although the SAD shape remained largely unchanged, the identity of the dominant species tended to vary within and among grassland types. Our study shows that for a given taxonomic group, the SAD shape is similar across habitat types with low‐intensity disturbances resulting from different management. This suggests that SADs respond primarily to the intensity of disturbance and thus could be best used in monitoring communities across strong disturbance and environmental gradients. Because taxonomic groups can inherently have different SADs, taxon‐specific SADs for undisturbed communities must be empirically documented before the SAD shape can be used as an indicator of environmental change. Because the identity of the dominant species changes from management type to another, the SAD shape alone is not an adequate monitoring tool.

## 1 INTRODUCTION

Grasslands are species rich but threatened habitats globally (Hoekstra *et al*. 2005). Especially, meadows and pastures, which traditionally were maintained by low‐intensity haymaking and grazing, have become rare (Tscharntke *et al*. 2005). At the same time, human activities create and maintain biotopes that may act as compensatory or complementary habitats for grassland species. Considering species that traditionally inhabited meadows and pastures, road verges have been shown to be such habitat types (Munguira & Thomas 1992; Cousins 2006). To enhance grassland biodiversity, we must document the patterns in different types of grassland communities and evaluate how management influences these communities. In this study, we investigated how low‐intensity management affects composition and structure of communities in pastures, meadows, and road verges.

Habitat management, or land‐use broadly, is a disturbance factor having diverse effects on community composition and diversity, depending on the type, intensity, and frequency of actions. In pastures, the main disturbance factor is livestock grazing. Grazing is more continuous and selective disturbance than mowing and removes vegetation closer to the ground (Rook *et al*. 2004). Animal activities, such as trampling and feces, have also additional effects on the environment (Kohler *et al*. 2006; Bilotta *et al*. 2007). In meadows and road verges, the main disturbance factor is mowing. In Finland, meadows and road verges are mowed a few times annually but with different methodology: Meadows are hand‐mowed, road verges machine‐mowed. Historically, the most productive soils were reserved for agriculture, and the remaining areas were used as meadows and pastures, often by turns. Thus, one can assume that the biotas in meadows and pastures were originally somewhat similar and have remained so, at least in comparison with the constructed road verges. Taken together, all these differences may affect the composition and structure of biological communities in meadows, pastures, and road verges.

The structure of biological communities can be characterized with species abundance distributions (SAD), which illustrate how the total number of individuals in a community is divided among species. Virtually, all SADs include a few dominant and many rare species (McGill *et al*. 2007; but see Dornelas & Connolly 2008). Understanding the causes and consequences of SADs is one of the oldest challenges in ecology (Raunkiaer 1909; Motomura 1932; Preston 1948), and still in research focus (Dornelas *et al*. 2009; Barabás *et al*., 2013; Locey & White 2013; Saether *et al*., 2013; Matthews & Whittaker 2015). Nevertheless, it is not well understood how SADs respond to natural or human‐induced environmental gradients, or to different types of land‐use and management (McGill *et al*. 2007; Dornelas *et al*. 2009; Simons *et al*. 2015). Furthermore, the SAD shape alone does not provide an adequate description of community patterns, especially as the shape may remain unchanged, while species composition or species rank abundance position (e.g., dominant species) changes in response to environmental changes. Hence, including species identity to SADs (i.e., the labeled SAD *sensu* McGill *et al*. 2007) allows for more thorough evaluation of the changes in community patterns.

The SAD approach has several benefits in comparison with other diversity measures (e.g., species richness or diversity indices). Most importantly, SADs help to identify and quantify changes in different parts of the community (e.g., among the dominant or rare species) and thus provide a tool for better understanding of the mechanisms behind community changes (McGill *et al*. 2007; Matthews & Whittaker 2015). Moreover, changes in the SAD shape itself can be easier to monitor than changes in species abundances (Arellano *et al*. 2017). Although the typical hollow‐curve shape of SADs has been criticized to result from statistical, rather than from ecological mechanisms (Yen *et al*., 2013), it has been repeatedly demonstrated that SADs vary in response to environmental factors (e.g., Simons *et al*. 2015; Arellano *et al*. 2017). There has also been a recent change in the research outlook; rather than testing a plethora of statistical models and searching for the best fit, the alternative approach to analyze how different SAD properties (e.g., skewness) vary with different predictor variables has gained popularity (Matthews & Whittaker 2014; Simons *et al*. 2015). This has better allowed for quantitative comparison of community changes across habitat types, land‐use intensities, or disturbance gradients (Dornelas *et al*. 2009; Yen *et al*. 2013; Simons *et al*. 2015), and the use of SADs in applied ecology has increased (Matthews & Whittaker 2015).

Generally, increasing disturbance intensity and frequency increases the dominance of disturbance‐tolerant species and makes SADs steeper, which has been demonstrated for plant and animal communities (Chaneton & Facelli 1991; Di Giulio *et al*. 2001; Kitahara & Sei 2001; Simons *et al*. 2015, 2017). Furthermore, different types of disturbance and management may have divergent effects on SADs (Simons *et al*. 2015, 2017; Chisté *et al*. 2016). Different management types can also affect different parts of the SAD; for example, fertilization can drive changes in dominance, whereas grazing and mowing can drive changes in the number of rare species (Simons *et al*. 2015). We investigated changes in the SADs of insect communities in three grassland types (meadows, pastures, road verges), which differed in their low‐intensity management modes (hand mowing, grazing, machine mowing, respectively). Our study taxa (Heteroptera, Carabidae, and Formicidae) differ greatly in their species richness and abundance and reflect differences in trophic position: Heteroptera are largely herbivores, Carabidae are predators, and Formicidae are omnivores. We asked (1) does the shape and composition of SADs differ among the grassland types, (2) does the shape of SADs change with increasing environmental heterogeneity (measured as plant species richness) or increasing disturbance (measured as trampling in pastures), and (3) are these changes in SADs consistent among taxa. Because grazing induces a continuous disturbance, we expect that the SAD should be steeper (less even) in pastures than in meadows and road verges. Partly for the same reason, SADs should also become steeper with increasing trampling and decreasing plant species richness. Given the differences in the ecology of the studied taxa, we expect that the identity of the dominant species should vary among the grassland types, and some of the changes in the SAD shape should be taxon‐specific.

## 2 MATERIALS AND METHODS

### 2.1 Study sites

This study was conducted in the southern and middle boreal vegetation zone in Central Finland. The region is forest dominated, and the total area of meadows and pastures is only 0.04% (742 ha) of the total land area. The extent of the study area was 115 km N‐S and 75 km E‐W. We practically selected all the traditional rural biotopes (TRBs) which met the following criteria: They had to be (1) classified as locally, regionally, or nationally valuable sites in the Finnish inventory of TRBs in the 1990s (Vainio *et al*. 2001); (2) ≥0.2 hectares; (3) mesic or dry meadows; and (4) managed by grazing or mowing for some decades and still under management. Ultimately, we included 12 meadows and 12 pastures in the study (Table S1), and these were paired spatially with each other (minimum and maximum distances between the pairs were 50 m and 64 km, respectively). The mean ± *SD* area of the pastures and meadows was 5.8 ± 9.0 ha, and they were surrounded by forests and grasslands. The most common (occurred in most sites) plant species were *Veronica chamaedrys*,* Agrostis capillaris, Alchemilla* sp., *Festuca rubra*,* Fragaria vesca*,* Hypericum maculatum*,* Poa pratensis*,* Ranunculus acris*,* Rumex acetosa,* and *Taraxacum* spp.; these were almost equally common in meadows and pastures. None of the sites were fertilized.

We selected road verges in‐between or nearby the meadow–pasture pairs. We a priori chose the nearest road from the map that met the following criteria: The road had to be (1) local tarmacked road or bigger according to the Finnish road classification to ensure at least 3‐m wide verges, and (2) built >20 years ago to allow grassland vegetation time to develop (for the same reason, no visible renovation actions were allowed); the selected roads were all different. After selecting the road, we drove from the predetermined starting point and selected the first suitable site. This was carried out in mid‐May so there was practically no green vegetation to influence site selection. To make the road verges comparable with pastures and meadows, we avoided the very moist and dry verges. All the verges were on the south or southeast side of the road and bordered by forest from this side. The most common plant species were *Achillea millefolium, Festuca rubra, Taraxacum* spp., *Epilobium angustifolium, Hieracium umbellatum, Hieracium vulgata* group, *Trifolium repens, Anthriscus sylvestris, Betula pubescens,* and *Cerastium fontanum*.

### 2.2 Species sampling

In each study site, we placed five 2 × 2 m sample quadrats in 10‐m intervals along a randomly placed 50 m transect; in meadows and pastures, the quadrats were at least 5 m from the forest edge. In small sites, we divided the transect in two, such that the second transect run perpendicularly to the first one. At the road verges, quadrat edge was at least one meter from the tarmac. To evaluate whether the different grassland types vary in their abiotic conditions, soil samples were taken from the corners of the quadrats and analyzed for soil fractions, pH, soil organic matter, and soil moisture (Table S1). All vascular plant species from the five quadrats were identified during June and July 2014. Plant species richness did not differ among the three habitat types (χ^2^ = 1.70, *df* = 2, *p* = .43). In pastures, the intensity of trampling (proportion of soil cover disturbed by grazers) and grazing (proportion of vascular plant shoots >5 cm in height that had been snapped off) was measured for each plot during three visits (in the turn of June–July, in August, and in the turn of September–October) and the average of these was used as an indicator of trampling and grazing intensity. We used the average from these three visits, instead of the first one only which coincided with the insect sampling, because it is likely to give a better estimate of the full‐season grazing and trampling pressure; in general, insects’ abundance and distribution in a given season are influenced by the animal activities in the previous season. Because trampling and grazing intensities were highly correlated (*r* = .842, *n* = 12, *p* < .001; Table S1), we selected only trampling intensity for further analyses.

For ground‐dwelling Heteroptera, Carabidae, and Formicidae, we used pitfall traps (200 ml, 6.5 cm diameter). Two pitfall traps were placed in the opposite corners of each quadrat, that is, there were 10 pitfall traps in each site (36 sites × 10 pitfalls = 360 traps altogether). We covered all pitfall traps with a plywood roof (2 cm above ground) to exclude rainwater. In a few sites, some pitfall traps were partly destroyed by cattle and by road maintenance. We did not try to compensate for the missing data, because the SAD shape is rather robust against variation in sampling intensity (Matthews & Whittaker 2015) and it is poorly known how sampling affects the SAD (McGill *et al*. 2007). To obtain a comprehensive view of the heteropteran community, we used two additional sampling methods: window trapping and sweep netting. In each site, one window trap (yellow bowl with diameter = 35 cm, window height 50 cm) was set up on ground to catch flying Heteroptera. Traps were filled with saltwater to preserve the material and with soap to reduce surface tension. Traps were set up 26–30 May 2014 and emptied twice (18—22 June and 7—11 July) such that they all were catching equal time periods. In 6–11 July, we used sweep netting (150 sweeps per site taken along the 50 m transect) to catch vegetation associated Heteroptera. To better enhance comparison among the grassland types, we sampled each triplet (meadow, pasture, road verge) on the same day. Adult specimens were identified and the nomenclature mainly follows Rintala & Rinne (2010), Lindroth (1986), and Douwes *et al*. (2012). *Formica rufa*,* F. lugubris*,* F. aquilonia,* and *F. pratensis* were considered as the *Formica rufa* group (Douwes *et al*. 2012).

### 2.3 Statistical analyses

We used three characteristics of SADs to interpret changes in their shape: abundance decay rate (*r*), dominance (*d*), and relative number of rare species, that is, rarity (Fisher's α/species richness) (Simons *et al*. 2015). Abundance decay rate (*r*) is the fitted parameter of the geometric series model (i.e., niche pre‐emption model; Motomura 1932) for SADs. In the geometric series model, the expected abundance of a species is defined as

$$n_{i}=N\;C\;r{\left(1-r\right)}^{i-1}\;\mathrm{with}\;C={\left[1-\left(1-r\right)S\right]}^{-1}$$

where *N* is the total number of individuals, *r* is the estimated decay rate per rank, and *S* is the total number of species (Magurran 2004). Thus, *r* describes the overall steepness of the SAD curve. We estimated it with the function “rad.preempt” in R package “vegan” (Oksanen *et al*. 2012). Dominance *d* which is also known as Berger‐Parker *d* (May 1975) is simply *d *= *N1/N* where *N1* is the number of individuals of the most abundant species, and *N* is the total abundance of all species. Fisher's α is an implicit function of the Fisher's log‐series distribution parameter and total community abundance (Fisher *et al*. 1943). It describes the number of rare species in a community (Magurran 2004). We estimated Fisher's α by fitting the log‐series model with the function “fisherfit” in package “vegan” (Oksanen *et al*. 2012). Fisher's α was further divided by a total number of species (*S*) to get the relative number of rare species (α/*S*).

We estimated the three parameters for each of the 36 communities, separately for Carabidae, Formicidae, and Heteroptera. For Heteroptera, parameters were estimated separately for the pitfall trap, window trap, and sweep net samples. To interpret the importance and sign of explanatory variables on the SAD shape (*r*,* d*, α/*S*), we developed a complete set of 16 linear models. These models included all combinations of explanatory variables (species richness, habitat type, plant species richness) and interactions between habitat type and plant species richness, as well as an intercept only model. We also included species richness in the models because it is expected to affect the SAD shape; although species richness is included in one of the explanatory variables (α/S, proportion of rare species), this may still be affected by species richness. We logit‐transformed *r* and *d* values prior to analyses because they are restricted between 0 and 1.

We used information theoretic approach, namely AICc, to compare the alternative models: The model with the smallest AICc is considered to be best with respect to expected Kullback–Leibler information lost developed for small sample sizes (Burnham & Anderson 2002). As the best model is not always apparent, we calculated model‐averaged parameter estimates of each variable using the models for which *Δ*_*i*_ **< 4 (*Δ*_*i*_ = AICc_min_ − AICc_i_) (Burnham & Anderson 2002). In one site, window trapping yielded no Heteroptera and in three sites all the observed species had exactly the same number of individuals producing meaningless α values. The latter was also true with pitfall trapping. We excluded these cases from the analyses of α/S resulting *n *=* *32 for window trapping and *n *=* *33 for pitfall trapping. Trampling was only analyzed in pastures (*n *=* *12). Again, for the above‐mentioned reason, *n *=* *11 in the α/S analyses for the window and pitfall trapped Heteroptera. We developed a set of eight linear models including all combinations of explanatory variables (species richness, plant species richness, trampling intensity) and compared them to each other and to a model containing intercept only on the basis of AICc. We conducted analyses with R version 3.3.2.

Differences in the number of species and individuals among the grassland types were analyzed with generalized linear models, using IBM SPSS Statistics 24. Because the response variable was count data, we used a Poisson distribution. In the analyses of the number of individuals, the model assumptions were not met, so we used a negative binomial distribution, which is more conservative in terms of Type I error. Dispersion parameter of the negative binomial distribution was estimated from the data with maximum likelihood.

## 3 RESULTS

Altogether, we recorded 96,340 insect specimens belonging to 203 species: 2,734 individuals and 113 species of Heteroptera, 90,347 individuals and 24 species of Formicidae, and 3,259 individuals and 66 species of Carabidae (Table S2). There was more heteropteran species in meadows than in road verges (χ^2^ = 8.46, *df* = 2, *p* = .015; mean difference = 4.48, CI_95%_ = 1.50–7.47, *p* = .003) but no difference in the number of individuals (χ^2^ = 3.53, *df* = 2, *p* = .171). For Carabidae and Formicidae, there were no differences in the average number of species or individuals between pastures, meadows, and road verges (χ^2^ < 4.44, *df* = 2, *p* > .15).

**Figure 1:**
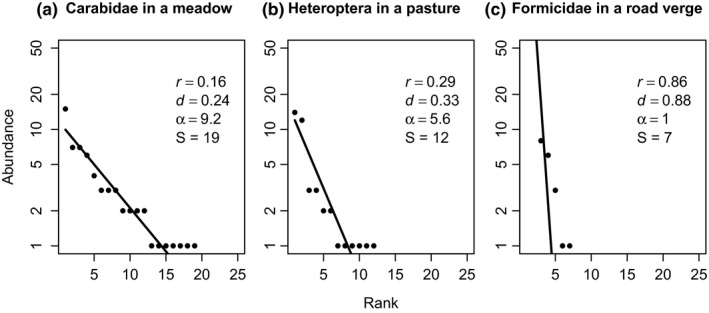
Three examples of the fitted geometric series models (line) and the values for the abundance decay rate (*r*), dominance (*d*), Fisher's alpha (α), and species richness (S). For Heteroptera, data pooling the three sampling methods (sweep netting, window, and pitfall trapping) are shown

**Figure 2:**
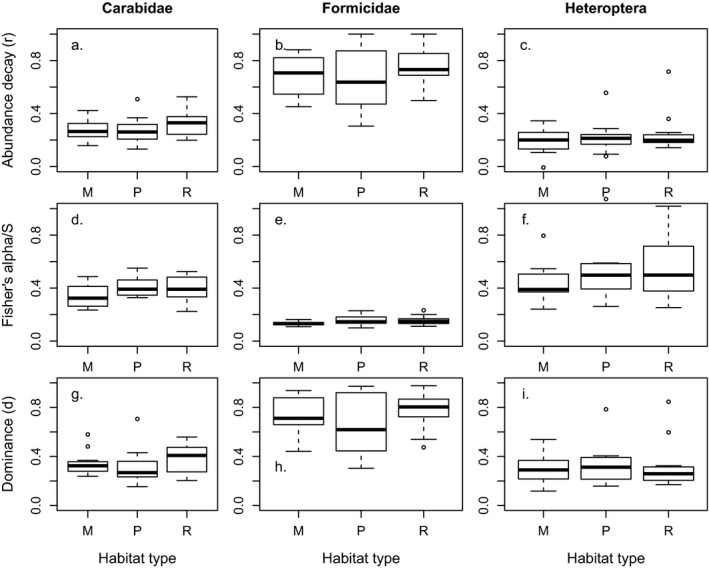
Abundance decay rate (*r*), dominance (*d*), and Fisher's α divided by the number of species (S) of Carabidae (a, d, g), Heteroptera (b, e, h), and Formicidae (c, f, i) do not differ among meadows (M), pastures (P), and road verges (R). The bottom and top of the box represent the 25th and 75th percentiles, respectively, and the horizontal bar represents the median. For Heteroptera data pooling, the three sampling methods (sweep netting, window, and pitfall trapping) are shown

Shapes of the SADs were very diverse, ranging from steep with few rare species to shallow with a long “tail” of singletons (Figure 1). The studied taxa showed differences in the SAD shape: decay rates (*r*) and dominance (*d*) were larger for Formicidae than for Carabidae and Heteroptera, whereas rarity (α/S) was smaller for Formicidae than for Carabidae and Heteroptera (Figure 2).

Species richness of the taxa itself significantly decreased the abundance decay rates (*r*) and dominances (*d*) for Carabidae and Heteroptera. The more plant species, the larger was rarity (α/S) for Carabidae and sweep‐netted Heteroptera (Tables 1 and S3). When plant species richness had a significant effect on rarity (α/S), also the species richness of a taxon had a negative effect (Table 2). For Formicidae, plant species richness affected none of the SAD characteristics: The intercept only model was considered the best one for all measures (Table S3). However, for Formicidae, dominance (*d*) increased with increasing trampling (Tables 2 and S4). For other taxa, the effect of trampling was negligible (Tables 2 and S4).

**Table 1:** Model‐averaged parameter estimates (from models with *Δ*_*i*_ **< 4) for explanatory variables (meadow is used as a baseline for the other habitat types) on abundance decay rate (*r*), dominance (*d*), and rarity (Fisher's α/S) in different taxa and sampling methods (*n* = 36). The parameter estimate is shown in bold if 95% CIs do not encompass zero

	Species richness			Plant species richness			Pasture			Road verge		
	Coeffi.	95% CI		Coeffi.	95% CI		Coeffi.	95% CI		Coeffi.	95% CI	
Carabidae												
*r*	**−0.063**	**−0.09**	**−0.035**	**−**0.012	**−**0.025	0.000						
*d*	**−0.059**	**−0.097**	**−0.022**	**−**0.003	**−**0.021	0.014	**−**0.256	**−**0.666	0.154	**−**0.035	**−**0.463	0.394
α/S	**−0.008**	**−0.014**	**−0.003**	**0.005**	**0.002**	**0.007**	0.055	**−**0.001	0.110	0.051	**−**0.008	0.110
Formicidae												
*r*	0.066	**−**0.140	0.272	**−**0.019	**−**0.059	0.022	0.106	**−**0.865	1.076	0.497	**−**0.473	1.468
*d*	0.125	**−**0.092	0.341	**−**0.027	**−**0.069	0.016	**−**0.112	**−**1.151	0.927	0.390	**−**0.649	1.429
α/S	**−**0.001	**−**0.007	0.004	0.000	**−**0.001	0.001	0.023	**−**0.003	0.049	0.023	**−**0.003	0.049
Heteroptera sweep netting												
*r*	**−0.126**	**−0.214**	**−0.038**	**−**0.008	**−**0.036	0.020						
*d*	**−0.116**	**−0.214**	**−0.019**	**−**0.010	**−**0.041	0.021						
α/S	**−0.067**	**−0.131**	**−0.003**	**0.023**	**0.003**	**0.044**						
Heteroptera pitfall trapping												
*r*	**−0.221**	**−0.351**	**−0.092**	**−**0.008	**−**0.035	0.020						
*d*	**−0.192**	**−0.336**	**−0.048**	**−**0.012	**−**0.043	0.018						
α/S*	**−**0.015	**−**0.100	0.070	**−**0.009	**−**0.027	0.008	0.205	**−**0.196	0.607	0.245	**−**0.157	0.647
Heteroptera window trapping												
*r*	**−0.216**	**−0.310**	**−0.122**	**−**0.039	**−**0.076	**−**0.003						
*d*	**−0.186**	**−0.284**	**−0.089**	**−**0.039	**−**0.077	**−**0.001						
α/S**	**−**0.033	**−**0.116	0.049	**−**0.002	**−**0.035	0.032	0.007	**−**0.771	0.784	0.445	**−**0.352	1.242

*n = 33; ** *n* = 32.

**Table 2:** Model‐averaged parameter estimates (from models with *Δ*_*i*_ **< 4) for explanatory variables (species richness, plant species, and amount of trampling) on abundance decay rate (*r*), dominance (*d*), and rarity (Fisher's α/S) in pastures (*n* = 12). The parameter estimate is shown in bold if 95% CIs do not encompass zero

	Species richness			Number of plant species			Amount of trampling		
	Coefficient	95% CI		Coefficient	95% CI		Coefficient	95% CI	
Carabidae									
* r*	**−0.09**	**−0.119**	**−0.061**	**−0.026**	**−0.047**	**−0.005**			
* d*	**−0.099**	**−0.159**	**−0.039**	**−**0.02	**−**0.069	0.029			
* *α/S	**−0.007**	**−0.012**	**−0.002**	**0.008**	**0.004**	**0.013**			
Formicidae									
* r*	0.355	**−**0.262	0.972	0.023	**−**0.139	0.186	0.044	**−**0.007	0.095
* d*	0.396	**−**0.258	1.051				**0.055**	**0.003**	**0.107**
* *α/S							**−**0.001	**−**0.003	0
Heteroptera sweep netting									
* r*				**0.075**	**0.013**	**0.137**			
* d*				**0.081**	**0.012**	**0.15**	**−**0.014	**−**0.039	0.011
* *α/S	**−**0.154	**−**0.33	0.023	0.017	**−**0.075	0.108	**−**0.003	**−**0.037	0.031
Heteroptera pitfall trapping									
* r*	**−**0.065	**−**0.247	0.117	0.013	**−**0.047	0.073	**−**0.011	**−**0.032	0.01
* d*	0.023	**−**0.179	0.225	0	**−**0.066	0.065	**−**0.014	**−**0.036	0.008
* *α/S*	**−**0.055	**−**0.252	0.143	**−**0.044	**−**0.097	0.009	0.013	**−**0.009	0.034
Heteroptera window trapping									
* r*	**−0.17**	**−0.305**	**−0.036**				**−**0.02	**−**0.042	0.003
* d*	**−**0.124	**−**0.318	0.07	0.021	**−**0.078	0.12	**−**0.025	**−**0.057	0.006
* *α/S*				0.077	**−**0.007	0.161	0.019	**−**0.014	0.052

*n = 11.

Although the shape of the SAD remained unchanged among the grassland types for Carabidae and Heteroptera, the identity of the dominant species varied among (Heteroptera) and within grassland types (Carabidae and Heteroptera). The most abundant Carabidae in all grassland types was *Pterostichus melanarius*, which comprised 22%, 14%, and 31% of the total carabid individuals in meadows, pastures, and road verges, respectively. The most abundant heteropteran species was *Plagiognathus chrysanthemi* (21%) in meadows, *Leptopterna dolabrata* (19%) in pastures, and *Chlamydatus pulicarius* (19%) in road verges. The most abundant Formicidae species was *Lasius platythorax* in meadows (42%) and in road verges (82%), and *Formica rufa* in pastures (75%). Altogether, 13 (20%) Carabidae, six (25%) Formicidae, and 14 (12%) Heteroptera species were dominant at least in one site, the most frequent species being *P. melanarius* (16 sites of which six were meadows, three pastures, and seven road verges), *L. platythorax* (22 sites of which six were meadows, seven pastures, and nine road verges), and *P. chrysanthemi* (nine sites of which four were meadows, four pastures, and one road verge), respectively.

## 4 DISCUSSION

In this paper, we characterized the empirical SADs for insect communities (Carabidae, Formicidae, and Heteroptera) in three grassland types (meadows, pastures, and road verges), which differed in their management. We further studied whether the SAD shape changes along with environmental heterogeneity (plant species richness) and disturbance (trampling intensity in pastures only), and whether these changes are consistent among different taxa and grassland types.

### 4.1 Differences among grassland types

Generally, the SAD shape did not vary among the grassland types. Thus, it seems that the different management regimes in our study did not create considerable variation in environmental conditions. This was supported also in that plant species richness, an indicator of environmental heterogeneity did not differ among the grassland types. Large proportion of the variation in the shape of SADs (especially in abundance decay rate and dominance) for Carabidae and Heteroptera was due to the variation in species richness of the taxon itself among sites. This emphasizes the intimate linkage between the SAD shape and species richness, as well as other macroecological patterns (e.g., species‐area relationship; McGill *et al*. 2007).

The type and frequency of disturbance were similar in meadows and road verges, and thus the similarity of the SAD shape between them was, to some extent, expected. By contrast, pastures are more disturbed habitat types than meadows and road verges because livestock grazing is more continuous than occasional mowing. Moreover, grazing is selective and removes vegetation closer to the ground (Rook *et al*. 2004), and trampling induces an additional disturbance type (Kohler *et al*. 2006). Thus, we expected that SADs should be steepest and have higher dominance in pastures. Contrary to our predictions, dominance was not higher in pastures than in the other two habitat types. One reason might be that grazing was not very intensive; it was mainly introduced to support biodiversity rather than to produce dairy or meat. Indeed, disturbance intensity is one key factor influencing SADs (Simons *et al*. 2015; Chisté *et al*. 2016). Due to pasture rotation, there have been short gaps in grazing in many of the sites, which in general should enhance grassland diversity, that is evenness of the SAD (Allan *et al*. 2014). Thus, although there was some variation in the type and intensity of disturbances between pastures and the other two grassland types, the overall disturbance intensity was rather low. Nevertheless, there were differences in the identity of the dominant species (see below).

The effect of disturbance intensity was studied only in pastures and it was measured as the amount of trampling. While trampling did not seem to affect Carabidae or Heteroptera, it increased dominance in Formicidae: Almost 40% of the variation in dominance was explained by the amount of trampling. This is in accordance with the other studies emphasizing the significance of disturbance intensity as a predictor of SAD characteristics (Gray 1979; Hill *et al*. 1995; Simons *et al*. 2015, 2017). If and when trampling intensity increased the dominance in Formicidae in pastures, why was there no difference between the pastures and the nongrazed grasslands? Statistically “no difference” means that there was no difference in the mean disturbance intensity between pastures and the nongrazed grassland types. Variation in trampling intensity among pastures, however, was large enough to document a response in Formicidae. Biologically, this result is plausible because mowing, as done in our system, is likely to mimic moderate (average) grazing intensity.

### 4.2 Differences among taxa

Variation in the SADs was considerable among the three taxa studied. Carabidae and Heteroptera had similar dominance and rarity pattern, as well as similar overall shape of the SAD. Formicidae, in turn, had much higher dominance, steeper decline, and lower rarity than Carabidae and Heteroptera. The results for Carabidae and Heteroptera agree well with those found in other studies (Komonen *et al*. 2015; Simons *et al*. 2015, 2017). Because the dominant species were all generalists, SAD differences among the three species groups cannot be explained by differences in specialization. Of course, one could have expected that Heteroptera, as herbivores, would have responded to changes in plant species richness more strongly than predatory Carabidae or omnivorous Formicidae. The difference between Formicidae and the other taxa is probably related to the smaller number of species, as well as to their colonial life style. This is supported in that very similar difference in dominance between Carabidae and Formicidae was observed in afforested fields (Komonen *et al*. 2015). Indeed, the abundance–distribution relationships of colonial species often diverge from the general macroecological patterns (Gaston & Blackburn 2000), and this seems to apply also to the SAD, as we demonstrate. Different taxa also showed variable responses to environmental heterogeneity. While Carabidae and Heteroptera seemed to be associated with plant species richness, Formicidae were not.

In general, disturbances increase the abundance of some disturbance‐tolerant species. The rationale is that disturbances kill individuals of different species unevenly, as well as affect the availability and spatio‐temporal distribution of resources (Chaneton & Facelli 1991; Di Giulio *et al*. 2001; Kitahara & Sei 2001; Simons *et al*. 2015). Carabidae seemed to respond rather uniformly to different disturbance types, because the dominant species *P. melanarius*, a predator inhabiting open areas (Lindroth 1986), was the same in all habitat types. Heteroptera, in turn, were dominated by different species in all habitat types: *P. chrysanthemi* in meadows, *L. dolabrata* in pastures, and *C. pulicarius* in road verges. All of the three species are generalists but feed on different plants: *P. chrysanthemi* Fabaceae and Asteracae on different types grasslands (Rintala & Rinne 2010), *L. dolabrata* mainly on Poaeceae, and *C. pulicarius* on many different grasses and herbs. For Formicidae, there were two dominant species: *L. platythorax* in meadows and road verges, and *F. rufa* in pastures, both of which occur in forests and forest edges (Douwes *et al*. 2012). So, although meadows and pastures were originally similar and still more natural habitat types than the constructed road verges, this is neither seen in the SAD shape nor in the identity of the dominant species. Thus, road verges could be viewed as complementary habitat for many of the studied taxa.

## 5 CONCLUSIONS

Species abundance distributions can be useful monitoring tools in nature conservation and land‐use management. In this paper, we demonstrate that low‐intensity, even though somewhat dissimilar, disturbances do not shape the SAD profoundly, and most of the variation in the SAD shape is due to the variation among taxa. Despite the similar SAD shape, the identity of the dominant species varies from one grassland type to another, as well as along the environmental gradients within grassland types; thus, the SAD shape alone is not an adequate monitoring tool. Although road verges could be seen as complementary habitat for insects in meadows and pastures, the observed taxa were mostly generalists. Thus, rather than indicating the good ecological quality of road verges, our data are more likely to indicate the poor quality of the studied meadows and pastures. In applied ecology, future studies on the SAD should focus on documenting and explaining variation in parameter estimates across different natural and human‐induced environmental gradients. We also urge more focus on the concomitant changes in species’ rank abundance position.

## CONFLICT OF INTEREST

None declared.

## AUTHORS' CONTRIBUTIONS

AK conceived the original idea, designed methodology, and collected the data; AK and ME developed the idea further, analyzed the data, and wrote the manuscript. Both authors contributed critically to the drafts and gave final approval for publication.

## DATA ACCESSIBILITY

The research data will be deposited and opened in the University of Jyväskylä Dataverse Network (https://dvn.jyu.fi/dvn).
