# Supplementary material for: Ecological response hides behind the species abundance distribution: Community response to low‐intensity disturbance in managed grasslands
Source: Ecol Evol. 2017 Sep 12;7(20):8558–66. doi: 10.1002/ece3.3395 (PMC5648673; doi:10.1002/ece3.3395)
Supplement: Supplementary file 1 [file ECE3-7-8558-s001.docx]

**Supporting Information**

Table S1. Summary of the study sites. Area is the area of the given administrative unit. Soil fractions and pH were recorded from the soil core samples that were taken from each of the five sample quadrats and pooled in one composite sample for each site. Only pH differed among the habitat types, being higher (5.25) in road verges than in meadows (4.63) or pastures (4.41) (Χ^2^ = 34.51, df = 2, p < 0.001), whereas soil organic matter and soil moisture did not differ (Χ^2^ < 1.47, df = 2, p > 0.48). There was no difference in the average size of the studied meadows (4.3 ha) and pastures (7.3 ha) (Χ^2^ = 0.71, df = 1, p = 0.40; it was not meaningful to calculate areas for road verges). Soil fractions were mainly silt in meadows and pastures, and sand in road verges.

| **Site*** | **Area ha** | **Soil fractions** | **pH** | **Grazing %** | **Trampling %** | **Plant species richness** |
| --- | --- | --- | --- | --- | --- | --- |
|  |  |  |  |  |  |  |
|  |  |  |  |  |  |  |
| **Meadows** |  |  |  |  |  |  |
| Hettee | 2 | fine sand | 4.45 | 0 | 10 | 53 |
| Riihimäki | 0.9 | medium silt | 4.61 | 0 | 0 | 38 |
| Karhukorpi | 1 | fine sand | 4.62 | 0 | 0 | 35 |
| Vuorela | 0.5 | coarse silt | 4.6 | 0 | 0 | 46 |
| Liehu | 0.5 | coarse silt | 4.16 | 0 | 0 | 64 |
| Syysniemi | 0.4 | coarse silt | 4.62 | 0 | 0 | 40 |
| Vaaru | 0.4 | medium silt | 4.33 | 0 | 0 | 38 |
| Kirkonmäki | 0.3 | fine sand | 4.98 | 0 | 0 | 47 |
| Vaateri | 1.1 | coarse silt | 4.67 | 0 | 0 | 46 |
| Mäentalo | 11.9 | fine silt | 4.93 | 0 | 0 | 30 |
| Harju | 32.5 | medium silt | 4.39 | 0 | 9 | 57 |
| Peurala | 0.1 | coarse silt | 5.16 | 0 | 9 | 34 |
| **Pastures** |  |  |  |  |  |  |
| Kivijärvi (c) | 7.6 | coarse silt | 4.7 | 39 | 41 | 48 |
| Aatula (c) | 6.1 | medium silt | 3.73 | 74 | 35 | 49 |
| Mäentalo (s) | 11.9 | fine silt | 4.65 | 0 | 4 | 46 |
| Haapalehto (s,c) | 8.6 | coarse silt | 4.28 | 7 | 0 | 44 |
| Suuruspää (h) | 6 | fine silt | 4.98 | 57 | 37 | 41 |
| Ohramaa (c) | 5.2 | coarse silt | 4.16 | 7 | 17 | 59 |
| Nuuttila (h) | 3.1 | coarse silt | 4.7 | 62 | 43 | 34 |
| Huusko (h) | 2.6 | coarse silt | 3.96 | 77 | 38 | 45 |
| Pekkanen (c) | 1.7 | fine sand | 4.27 | 0 | 0 | 39 |
| Koivulahti (h) | 1.1 | fine sand | 4.24 | 50 | 14 | 43 |
| Harju (s) | 32.5 | medium silt | 4.07 | 37 | 10 | 37 |
| Vaateri (s) | 1.1 | coarse silt | 5.18 | 0 | 0 | 42 |
| **Road verges** |  |  |  |  |  |  |
| Myllypohja | na | coarse sand | 5.41 | 0 | 0 | 61 |
| Multia | na | coarse sand | 5.45 | 0 | 40 | 30 |
| Petäjävesi | na | coarse sand | 5.65 | 0 | 0 | 53 |
| Muurame | na | fine sand | 5.99 | 0 | 0 | 40 |
| Paateri | na | coarse sand | 5.66 | 0 | 0 | 20 |
| Rotkola | na | coarse sand | 4.75 | 0 | 0 | 54 |
| Klemettilä | na | coarse sand | 5.09 | 0 | 0 | 30 |
| Valospohja | na | coarse sand | 4.63 | 0 | 0 | 45 |
| Tammikoski | na | coarse sand | 5.14 | 0 | 0 | 30 |
| Jukola | na | coarse sand | 4.65 | 0 | 0 | 31 |
| Havulankangas | na | coarse sand | 5.22 | 0 | 0 | 44 |
| Norola | na | fine sand | 5.43 | 0 | 0 | 36 |

* c = cattle, s= sheep, h = horse

Table S2 Total number of individuals and species caught per site (n = 36) for each taxon and sampling method.

|  | Total abundance | | | Number of species | | |
| --- | --- | --- | --- | --- | --- | --- |
|  | median | min | max | median | min | max |
| Carabidae | 69 | 17 | 297 | 14 | 6 | 24 |
| Formicidae | 1280 | 102 | 22981 | 8 | 4 | 12 |
| Heteroptera |  |  |  |  |  |  |
| total | 60 | 17 | 301 | 16 | 7 | 30 |
| sweep netting | 31 | 5 | 239 | 8 | 2 | 14 |
| pitfal trapping | 10 | 2 | 59 | 4 | 1 | 10 |
| window trapping | 9 | 0 | 66 | 6 | 0 | 17 |

Table S3 The linear models (for which *Δ* < 4) describing the association between abundance decay rate (*r*), dominance (*d*) and rarity (Fisher’s α/S) and explanatory variables (S = species richness, habitat type, plant species richness) in different taxa and sampling methods (n = 36). Intercept and estimated coefficients for the explanatory variables, the proportion of the explained variance (R^2^), degrees of freedom (df), loglikelihood (logLik), the small-sample-size corrected version of Akaike Information Criterion (AICc), *Δ* (AICc_i_-AICc_min_) and Akaike weights (*w*) for models are presented. The model with the smallest AICc is considered to be best. Symbol + denotes that a factor ‘habitat’ is included in the model. * n = 32, ** n = 33

| Taxon | (Int) | S | | Habitat | Plant S | R^2^ | df | logLik | AICc | *Δ* | *w* |
| --- | --- | --- | --- | --- | --- | --- | --- | --- | --- | --- | --- |
| **Carabidae** |  |  | |  |  |  |  |  |  |  |  |
| *r* | 0.438 | -0.061 | |  | -0.012 | 0.46 | 4 | -12.2 | 33.7 | 0.0 | 0.68 |
|  | -0.020 | -0.066 | |  |  | 0.40 | 3 | -14.2 | 35.2 | 1.5 | 0.32 |
|  |  |  | |  |  |  |  |  |  |  |  |
| *d* | 0.161 | -0.059 | |  |  | 0.24 | 3 | -24.2 | 55.1 | 0.0 | 0.66 |
|  | 0.284 | -0.058 | |  | -0.003 | 0.24 | 4 | -24.1 | 57.5 | 2.4 | 0.20 |
|  | 0.263 | -0.060 | | + |  | 0.28 | 5 | -23.1 | 58.2 | 3.1 | 0.14 |
|  |  |  | |  |  |  |  |  |  |  |  |
| α/S | 0.303 | -0.009 | |  | 0.005 | 0.41 | 4 | 46.7 | -84.2 | 0.0 | 0.56 |
|  | 0.242 | -0.007 | | + | 0.005 | 0.49 | 6 | 49.3 | -83.7 | 0.5 | 0.44 |
|  |  |  | |  |  |  |  |  |  |  |  |
| **Formicidae** |  |  | |  |  |  |  |  |  |  |  |
| *r* | 1.042 |  | |  |  | 0.00 | 2 | -55.8 | 115.9 | 0.0 | 0.45 |
|  | 1.854 |  | |  | -0.019 | 0.03 | 3 | -55.3 | 117.3 | 1.4 | 0.22 |
|  | 0.497 | 0.069 | |  |  | 0.01 | 3 | -55.5 | 117.8 | 1.9 | 0.18 |
|  | 1.330 | 0.060 | |  | -0.018 | 0.04 | 4 | -55.1 | 119.5 | 3.6 | 0.08 |
|  | 0.841 |  | | + |  | 0.04 | 4 | -55.1 | 119.5 | 3.6 | 0.07 |
|  |  |  | |  |  |  |  |  |  |  |  |
| *d* | 1.240 |  | |  |  | 0.00 | 2 | -58.2 | 120.7 | 0.0 | 0.34 |
|  | 2.409 |  | |  | -0.028 | 0.05 | 3 | -57.3 | 121.3 | 0.6 | 0.25 |
|  | 0.215 | 0.130 | |  |  | 0.04 | 3 | -57.4 | 121.5 | 0.9 | 0.22 |
|  | 1.387 | 0.117 | |  | -0.025 | 0.08 | 4 | -56.6 | 122.5 | 1.9 | 0.14 |
|  | 1.147 |  | | + |  | 0.03 | 4 | -57.6 | 124.5 | 3.8 | 0.05 |
|  |  |  | |  |  |  |  |  |  |  |  |
| α/S | 0.148 |  | |  |  | 0.00 | 2 | 73.3 | -142.3 | 0.0 | 0.34 |
|  | 0.132 |  | | + |  | 0.12 | 4 | 75.6 | -141.9 | 0.4 | 0.28 |
|  | 0.159 | -0.001 | |  |  | 0.01 | 3 | 73.5 | -140.2 | 2.1 | 0.12 |
|  | 0.145 |  | |  | 0.000 | 0.00 | 3 | 73.3 | -139.9 | 2.4 | 0.10 |
|  | 0.139 |  | | + | -0.001 | 0.12 | 5 | 75.7 | -139.3 | 3.0 | 0.08 |
|  |  |  | |  |  |  |  |  |  |  |  |
| **Heteroptera sweep netting** | | | |  |  |  |  |  |  |  |  |
| *r* | 0.522 | | -0.126 |  |  | 0.20 | 3 | -42.0 | 90.8 | 0.0 | 0.75 |
|  | 0.855 | | -0.126 |  | -0.008 | 0.21 | 4 | -41.9 | 93.0 | 2.2 | 0.25 |
|  |  | |  |  |  |  |  |  |  |  |  |
| *d* | 0.696 | | -0.116 |  |  | 0.15 | 3 | -45.9 | 98.5 | 0.0 | 0.65 |
|  | 1.118 | | -0.116 |  | -0.010 | 0.16 | 4 | -45.7 | 100.6 | 2.1 | 0.23 |
|  | -0.242 | |  |  |  | 0.00 | 2 | -48.8 | 101.9 | 3.4 | 0.12 |
|  |  | |  |  |  |  |  |  |  |  |  |
| α/S | 0.276 | | -0.067 |  | 0.023 | 0.24 | 4 | -29.9 | 69.0 | 0.0 | 0.65 |
|  | -0.263 | |  |  | 0.023 | 0.13 | 3 | -32.3 | 71.3 | 2.2 | 0.21 |
|  | 1.269 | | -0.067 |  |  | 0.11 | 3 | -32.7 | 72.1 | 3.1 | 0.14 |
|  |  | |  |  |  |  |  |  |  |  |  |
| **Heteroptera window trapping** | | | |  |  |  |  |  |  |  |  |
| *r* | 2.716 | -0.211 | |  | -0.039 | 0.51 | 4 | -48.5 | 106.3 | 0.0 | 0.76 |
|  | 1.188 | -0.233 | |  |  | 0.43 | 3 | -50.9 | 108.6 | 2.3 | 0.24 |
|  |  |  | |  |  |  |  |  |  |  |  |
| *d* | 2.760 | -0.180 | |  | -0.039 | 0.43 | 4 | -49.9 | 109.1 | 0.0 | 0.72 |
|  | 1.239 | -0.202 | |  |  | 0.35 | 3 | -52.1 | 111.0 | 1.9 | 0.28 |
|  |  |  | |  |  |  |  |  |  |  |  |
| α/S* | 1.226 |  | |  |  | 0.00 | 2 | -41.1 | 86.5 | 0.0 | 0.52 |
|  | 1.451 | -0.033 | |  |  | 0.02 | 3 | -40.7 | 88.3 | 1.7 | 0.22 |
|  | 1.305 |  | |  | -0.002 | 0.00 | 3 | -41.1 | 89.0 | 2.4 | 0.16 |
|  | 1.084 |  | | + |  | 0.05 | 4 | -40.2 | 89.8 | 3.3 | 0.10 |
|  |  |  | |  |  |  |  |  |  |  |  |
| **Heteroptera pitfall trapping** | | | |  |  |  |  |  |  |  |  |
| *r* | 0.951 | -0.219 | |  |  | 0.26 | 3 | -39.9 | 86.5 | 0.0 | 0.75 |
|  | 1.317 | -0.228 | |  | -0.008 | 0.27 | 4 | -39.7 | 88.7 | 2.2 | 0.25 |
|  |  |  | |  |  |  |  |  |  |  |  |
| *d* | 0.972 | -0.187 | |  |  | 0.17 | 3 | -43.6 | 94.0 | 0.0 | 0.71 |
|  | 1.554 | -0.202 | |  | -0.012 | 0.19 | 4 | -43.3 | 95.8 | 1.8 | 0.29 |
|  |  |  | |  |  |  |  |  |  |  |  |
| α/S** | 0.708 |  | |  |  | 0.00 | 2 | -20.7 | 45.8 | 0.0 | 0.45 |
|  | 1.092 |  | |  | -0.009 | 0.04 | 3 | -20.1 | 47.0 | 1.2 | 0.24 |
|  | 0.768 | -0.013 | |  |  | 0.00 | 3 | -20.6 | 48.1 | 2.3 | 0.14 |
|  | 0.558 |  | | + |  | 0.06 | 4 | -19.7 | 48.9 | 3.1 | 0.10 |
|  | 1.200 | -0.019 | |  | -0.010 | 0.04 | 4 | -20.0 | 49.4 | 3.6 | 0.07 |

Table S4 The linear models (for which *Δ* < 4) describing the association between abundance decay rate (*r*), dominance (*d*) and rarity (Fisher’s α/S) and explanatory variables (S = species richness, plant species richness, amount of trampling) in different taxa and sampling methods only in pastures (n = 12). Intercept and estimated coefficients for the explanatory variables, the proportion of the explained variance (R^2^), degrees of freedom (df), loglikelihood (logLik), the small-sample-size corrected version of Akaike Information Criterion (AICc), *Δ* (AICc_i_-AICc_min_) and Akaike weights (*w*) for models are presented. The model with the smallest AICc is considered to be best. * n = 11

| Taxon | (Int) | S | Plant S | Trampling | R^2^ | df | logLik | AICc | *Δ* | *w* |
| --- | --- | --- | --- | --- | --- | --- | --- | --- | --- | --- |
| **Carabidae** |  |  |  |  |  |  |  |  |  |  |
| *r* | 1.352 | -0.089 | -0.026 |  | 0.88 | 4 | 4.0 | 5.8 | 0.00 | 0.80 |
|  | 0.249 | -0.092 |  |  | 0.78 | 3 | 0.3 | 8.5 | 2.70 | 0.21 |
|  |  |  |  |  |  |  |  |  |  |  |
| *d* | 0.566 | -0.100 |  |  | 0.58 | 3 | -6.5 | 22 | 0.00 | 0.86 |
|  | 1.411 | -0.097 | -0.020 |  | 0.61 | 4 | -6.0 | 25.6 | 3.62 | 0.14 |
|  |  |  |  |  |  |  |  |  |  |  |
|  | 0.135 | -0.007 | 0.008 |  | 0.74 | 4 | 23.1 | -32.4 | 0.00 | 0.83 |
| α/S | 0.058 |  | 0.008 |  | 0.50 | 3 | 19.1 | -29.3 | 3.16 | 0.17 |
| **Formicidae** |  |  |  |  |  |  |  |  |  |  |
| *r* | 0.068 |  |  | 0.044 | 0.27 | 3 | -19.5 | 48 | 0.00 | 0.40 |
|  | 0.947 |  |  |  | 0.00 | 2 | -21.4 | 48.1 | 0.08 | 0.38 |
|  | -1.955 | 0.355 |  |  | 0.14 | 3 | -20.5 | 49.9 | 1.92 | 0.15 |
|  | -0.078 |  | 0.023 |  | 0.01 | 3 | -21.3 | 51.6 | 3.62 | 0.07 |
|  |  |  |  |  |  |  |  |  |  |  |
| *d* | -0.095 |  |  | 0.057 | 0.38 | 3 | -19.3 | 47.6 | 0.00 | 0.56 |
|  | 1.035 |  |  |  | 0.00 | 2 | -22.2 | 49.8 | 2.15 | 0.19 |
|  | -2.900 | 0.482 |  |  | 0.22 | 3 | -20.7 | 50.4 | 2.76 | 0.14 |
|  | -2.183 | 0.280 |  | 0.047 | 0.45 | 4 | -18.7 | 51 | 3.39 | 0.10 |
|  |  |  |  |  |  |  |  |  |  |  |
| α/S | 0.182 |  |  | -0.001 | 0.38 | 3 | 25.6 | -42.3 | 0.00 | 0.74 |
|  | 0.155 |  |  |  | 0.00 | 2 | 22.8 | -40.2 | 2.06 | 0.26 |
|  |  |  |  |  |  |  |  |  |  |  |
| **Heteroptera sweep netting** | | |  |  |  |  |  |  |  |  |
| *r* | -3.661 |  | 0.075 |  | 0.42 | 3 | -9.8 | 28.5 | 0.00 | 0.81 |
|  | -0.367 |  |  |  | 0.00 | 2 | -13.0 | 31.4 | 2.86 | 0.19 |
|  |  |  |  |  |  |  |  |  |  |  |
| *d* | -3.608 |  | 0.081 |  | 0.40 | 3 | -11.1 | 31.2 | 0.00 | 0.65 |
|  | -0.062 |  |  |  | 0.00 | 2 | -14.2 | 33.7 | 2.50 | 0.19 |
|  | -3.376 |  | 0.082 | -0.014 | 0.49 | 4 | -10.1 | 33.9 | 2.76 | 0.16 |
|  |  |  |  |  |  |  |  |  |  |  |
| α/S | 1.962 | -0.154 |  |  | 0.27 | 3 | -12.6 | 34.1 | 0.00 | 0.45 |
|  | 0.937 |  |  |  | 0.00 | 2 | -14.5 | 34.3 | 0.16 | 0.41 |
|  | 0.205 |  | 0.017 |  | 0.02 | 3 | -14.4 | 37.8 | 3.63 | 0.07 |
|  | 0.998 |  |  | -0.003 | 0.00 | 3 | -14.5 | 37.9 | 3.77 | 0.07 |
|  |  |  |  |  |  |  |  |  |  |  |
| **Heteroptera window trapping** | | |  |  |  |  |  |  |  |  |
| *r* | 0.522 | -0.178 |  |  | 0.44 | 3 | -10.2 | 29.4 | 0.00 | 0.43 |
|  | 0.793 | -0.163 |  | -0.019 | 0.62 | 4 | -7.9 | 29.6 | 0.12 | 0.41 |
|  | -0.810 |  |  |  | 0.00 | 2 | -13.7 | 32.8 | 3.34 | 0.08 |
|  | -0.358 |  |  | -0.023 | 0.25 | 3 | -12.0 | 32.9 | 3.50 | 0.08 |
|  |  |  |  |  |  |  |  |  |  |  |
| *d* | -0.423 |  |  |  | 0.00 | 2 | -15.5 | 36.3 | 0.00 | 0.35 |
|  | 0.095 |  |  | -0.026 | 0.25 | 3 | -13.7 | 36.5 | 0.22 | 0.31 |
|  | 0.551 | -0.130 |  |  | 0.18 | 3 | -14.3 | 37.6 | 1.33 | 0.18 |
|  | 0.885 | -0.112 |  | -0.024 | 0.38 | 4 | -12.6 | 38.9 | 2.67 | 0.09 |
|  | -1.352 |  | 0.021 |  | 0.02 | 3 | -15.3 | 39.7 | 3.40 | 0.06 |
|  |  |  |  |  |  |  |  |  |  |  |
| α/S* | -2.365 |  | 0.077 |  | 0.33 | 3 | -10.5 | 30.4 | 0.00 | 0.47 |
|  | 1.091 |  |  |  | 0.00 | 2 | -12.6 | 30.8 | 0.41 | 0.39 |
|  | 0.750 |  |  | 0.019 | 0.16 | 3 | -11.7 | 32.8 | 2.40 | 0.14 |
|  |  |  |  |  |  |  |  |  |  |  |
| **Heteroptera pitfall trapping** | | |  |  |  |  |  |  |  |  |
| *r* | -0.069 |  |  |  | 0.00 | 2 | -9.5 | 24.3 | 0.00 | 0.57 |
|  | 0.151 |  |  | -0.011 | 0.12 | 3 | -8.7 | 26.5 | 2.12 | 0.20 |
|  | 0.208 | -0.065 |  |  | 0.06 | 3 | -9.1 | 27.3 | 2.93 | 0.13 |
|  | -0.651 |  | 0.013 |  | 0.02 | 3 | -9.4 | 27.7 | 3.38 | 0.11 |
|  |  |  |  |  |  |  |  |  |  |  |
| *d* | 0.178 |  |  |  | 0.00 | 2 | -10.4 | 26.1 | 0.00 | 0.56 |
|  | 0.449 |  |  | -0.014 | 0.16 | 3 | -9.4 | 27.7 | 1.58 | 0.26 |
|  | 0.080 | 0.023 |  |  | 0.01 | 3 | -10.4 | 29.7 | 3.59 | 0.09 |
|  | 0.190 |  | 0.000 |  | 0.00 | 3 | -10.4 | 29.8 | 3.67 | 0.09 |
|  |  |  |  |  |  |  |  |  |  |  |
| α/S* | 0.763 |  |  |  | 0.00 | 2 | -8.6 | 22.6 | 0.00 | 0.38 |
|  | 2.693 |  | -0.044 |  | 0.28 | 3 | -6.7 | 22.9 | 0.27 | 0.33 |
|  | 0.527 |  |  | 0.013 | 0.17 | 3 | -7.6 | 24.6 | 1.94 | 0.14 |
|  | 2.400 |  | -0.043 | 0.012 | 0.43 | 4 | -5.5 | 25.7 | 3.04 | 0.08 |
|  | 1.006 | -0.055 |  |  | 0.04 | 3 | -8.3 | 26.1 | 3.46 | 0.07 |
